# Effect of ouabain on calcium signaling in rodent brain: A systematic review of *in vitro* studies

**DOI:** 10.3389/fphar.2022.916312

**Published:** 2022-08-29

**Authors:** Jacqueline Alves Leite, Elisa Pôças, Gisele Silva Maia, Leandro Barbosa, Luis Eduardo M. Quintas, Elisa Mitiko Kawamoto, Maria Luiza Correia da Silva, Cristoforo Scavone, Luciana E. Drumond de Carvalho

**Affiliations:** ^1^ Departamento de Farmacologia, Instituto de Ciências Biológicas, Universidade Federal de Goiás, Goiânia, Brazil; ^2^ Campus Realengo, Instituto Federal do Rio de Janeiro, Rio de Janeiro, Brazil; ^3^ Laboratório de Bioquímica Celular, Universidade Federal de São João del Rei, Campus Centro-Oeste Dona Lindu, São Paulo, Brazil; ^4^ Laboratório de Farmacologia Bioquímica e Molecular, Instituto de Ciências Biomédicas, Universidade Federal do Rio de Janeiro, Rio de Janeiro, Brazil; ^5^ Departamento de Farmacologia, Instituto de Ciências Biomédicas, Universidade de São Paulo, São Paulo, Brazil

**Keywords:** ouabain, Na^+^/K^+^-ATPase, calcium, signaling, nervous system

## Abstract

The Na^+^/K^+^-ATPase is an integral membrane ion pump, essential to maintaining osmotic balance in cells in the presence of cardiotonic steroids; more specifically, ouabain can be an endogenous modulator of the Na^+^/K^+^-ATPase. Here, we conducted a systematic review of the *in vitro* effects of cardiotonic steroids on Ca^2+^ in the brain of rats and mice. Methods: The review was carried out using the PubMed, Virtual Health Library, and EMBASE databases (between 12 June 2020 and 30 June 2020) and followed the guidelines described in the Preferred Reporting Items for Systematic Reviews and Meta-analyses (PRISMA). Results: in total, 829 references were identified in the electronic databases; however, only 20 articles were considered, on the basis of the inclusion criteria. The studies demonstrated the effects of ouabain on Ca^2+^ signaling in synaptosomes, brain slices, and cultures of rat and mouse cells. In addition to the well-known cytotoxic effects of high doses of ouabain, resulting from indirect stimulation of the reverse mode of the Na^+^/Ca^2+^ exchanger and increased intracellular Ca^2+^, other effects have been reported. Ouabain-mediated Ca^2+^ signaling was able to act increasing cholinergic, noradrenergic and glutamatergic neurotransmission. Furthermore, ouabain significantly increased intracellular signaling molecules such as InsPs, IP3 and cAMP. Moreover treatment with low doses of ouabain stimulated myelin basic protein synthesis. Ouabain-induced intracellular Ca^2+^ increase may promote the activation of important cell signaling pathways involved in cellular homeostasis and function. Thus, the study of the application of ouabain in low doses being promising for application in neurological diseases.

**Systematic Review Registration:** https://www.crd.york.ac.uk/prospero/display_record.php?ID=CRD42020204498, identifier CRD42020204498.

## Introduction

Na^+^/K^+^-ATPase, also known as Na^+^-K^+^ pump, is a plasma membrane transporter essential for cellular homeostasis. It is responsible for the active movement of Na^+^ and K^+^ against their electrochemical gradients at the expenses of ATP hydrolysis, and supports the maintenance of the cellular osmotic balance, membrane potential and the secondary active transport of substrates and neurotransmitters (Blanco, 2005). Particularly in the brain, which is composed of highly specialized cells (i.e., neurons and glial cells), Na^+^/K^+^-ATPase is involved in neuronal excitability and astrocyte buffering of extracellular K^+^ ions during neuron action potential, in electrolyte balance of the cerebrospinal fluid, and in the secondary transport of molecules across the membrane, playing a fundamental role in the function of the central nervous system (Larsen et al., 2016), (Kinoshita et al., 2020). Therefore, the regulation of Na^+^/K^+^-ATPase function largely affects cell and system physiology.

**FIGURE 1:**
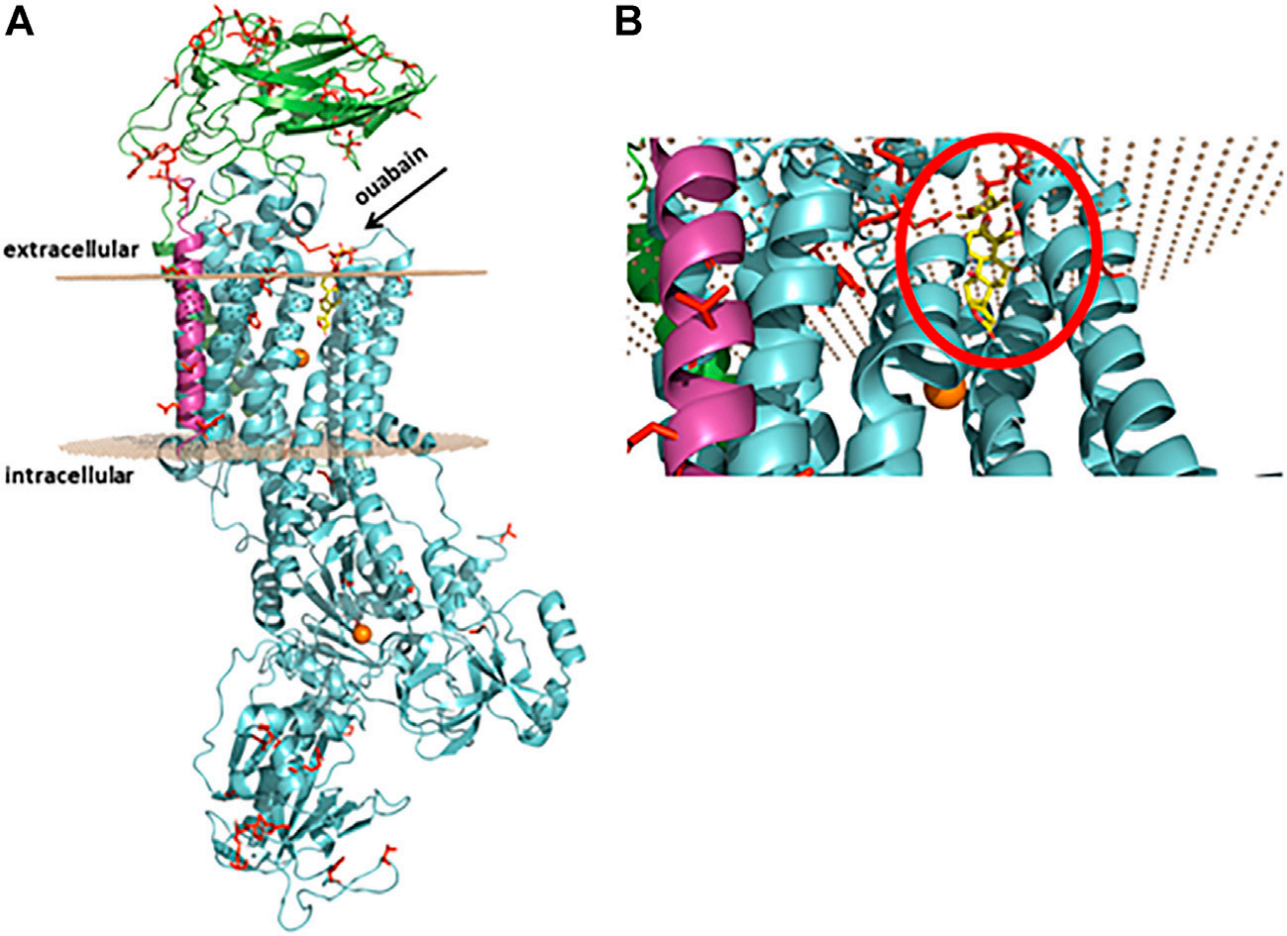
Protein representation of rat Na^+^/K^+^-ATPase α1β1γ isoform subunits. **(A)** In green, α1 subunit, in cyan, β1 subunit, and in pink, γ (FXYD2) subunit. The residues displaying the substitutions present in the rat compared to the human Na^+^/K^+^-ATPase protein. In orange, magnesium ions. The gray dots represent the Na^+^/K^+^-ATPase position at the plasma membrane, and the arrow points to the pocket where ouabain (in yellow) interacts. **(B)** Magnification of the binding pocket with ouabain (inside the red circle) seen from the bottom. The protein was constructed by homology using as reference the human crystallographic structure (pdb id:4RET) using the Swiss-model webserver, and for the representation the software PyMOL 7 was used.

This enzyme is composed of three subunits, and two of them are indispensable for enzyme activity (Figure 1). The amino acid sequence of the *α* subunit, also called the catalytic subunit, comprises more than 1010 residues (around 110 kDa) with 10 transmembrane domains. This subunit harbors the binding sites for ions (Na^+^, K^+^, Mg^2+^), ATP, and selective ligands collectively known as cardiotonic steroids, as well as for several other regulators (Blanco, 2005), (Blanco and Mercer, 1998) . All isoforms are encoded by different genes and present a high degree of homology (Clausen et al., 2017). An interesting feature is that Na^+^/K^+^-ATPase isoforms are expressed in a cell-/tissue-specific fashion (Blanco, 2005), (Cereijido et al., 2012), (Geering, 2008): the α1 isoform is ubiquitously expressed in mammalian tissues and, in rodents, it is 100–1000-fold less sensitive to cardiotonic steroids. In contrast, it has been shown not to be the case for the bufadienolide marinobufagenin (Fedorova and Bagrov, 1997), (Fedorova et al., 2001), although our group reported that it behaves similar to any known cardiotonic steroid (Godinho et al., 2017), (Carvalho et al., 2019); the α_2_ isoform is found in striated and smooth muscle, as well as in both astrocytes and neurons in the central nervous system, and adipose tissue; the α_3_ isoform is basically found in neurons but not in astrocytes and should be considered a neuronal marker (Dobretsov and Stimers, 2005); the α_4_ isoform is specifically found in the midpiece of sperm (Blanco, 2005). The *β* subunit is a type II glycoprotein of around 300 amino acids, that is, fundamental for a normal pumping activity. Enzyme kinetics modulation, Na^+^/K^+^-ATPase plasma membrane delivery and assembly, as well as cell adhesion and polarity, are characteristics of this subunit. The third subunit, *γ*, is a type I protein from the FXYD family (FXYD2) and it comprises around 65 amino acids. FXYD2 is not required for enzymatic activity but regulates Na^+^/K^+^-ATPase affinity for cations (Geering, 2008).

Ouabain is a natural ligand of Na^+^/K^+^-ATPase and belongs to the cardiotonic steroid class of compounds, i.e., the cardenolide family, originally identified in the African plants *Strophantus gratus* (Ouabain, 1932) and *Acokanthera ouabaio* (also called wabajo or schimperi) (Kupicha, 1982). Its inhibitory capacity was reported in 1953 by Schartzmann (Schatzmamn, 1953); this discovery paved the way for research on the mechanistic pathways of other cardiotonic steroids, such as digoxin, clinically used for decades to treat heart failure (Ziff and Kotecha, 2016). Ouabain selectively binds to Na^+^/K^+^-ATPase and inhibits its pump activity, increasing intracellular Na^+^ and Ca^2+^ concentrations ([Na^+^]i and [Ca^2+^]i, respectively), the latter by inducing a lower/reverse activity of the Na^+^/Ca^2+^ exchanger that colocalizes with the Na^+^/K^+^-ATPase (Larsen et al., 2016), (Kinoshita et al., 2020). In the heart, the indirect elevation of sarcoplasmic reticulum Ca^2+^ levels lead to the cardiac inotropism (Akera and Brody, 1977), (Lingrel, 2009). Higher concentrations of ouabain are known to evoke cardiotoxic effects. In other organs, such as the brain, the effects are more complex. Concentrations that induce bulk inhibition of Na^+^/K^+^-ATPase generate neurotoxic effects (Lees et al., 1990), (Veldhuis et al., 2003). In contrast, ouabain at a low concentration of 10 nm increases the expression of the brain-derived neurotrophic factor (BDNF) mRNA in cerebellar cell culture (de Sá Lima et al., 2013) or after intrahippocampal injection (Tata et al., 2014) and activates the Wnt/β-catenin signaling pathway culminating with the increase of CREB/BDNF and NF-κB levels (Kawamoto et al., 2012a), indicating that the balance between ouabain-induced neuroprotective and neurotoxic effects is concentration-dependent.

Several mechanisms are involved in cellular Ca^2+^ homeostasis. From Ca^2+^-binding proteins inside cells to proteins that allow Ca^2+^ fluxes across biological membranes, Ca^2+^ concentrations are strictly controlled in cytoplasm and organelles like mitochondria, Golgi apparatus, endoplasmic reticulum and nucleus (Brini et al., 2014). Ca^2+^ Channels (Koh et al., 2017), the high Ca^2+^ affinity but low capacity plasma membrane Ca^2+^-ATPases and the low Ca^2+^ affinity but high capacity Na^+^/Ca^2+^ exchanger (Blaustein et al., 2002), sarco/endoplasmic reticulum Ca^2+^-ATPases (Valadares et al., 2021), and ryanodine and inositol 1,4,5-triphosphate (IP3) receptors (McGarry and Williams, 1993), (Panizza et al., 2019) are important for Ca^2+^ handling and secondary targets for ouabain action.

Na^+^/K^+^-ATPase ion pumping is the classical function assigned to this transporter. Nevertheless, around 50 years after the discovery of this mechanism, a new paradigm emerged. A series of works performed by Zijian Xie and Amir Askari’s group unveiled that, besides ion transport, Na^+^/K^+^-ATPase operates as a receptor (Xie and Askari, 2002). The binding of ouabain to Na^+^/K^+^-ATPase triggers intracellular signaling networks through protein–protein interactions, generating a myriad of effects independent of the impairment of electrochemical gradients (Riganti et al., 2011). In this case, ouabain (and other cardiotonic steroids) would act as an agonist and not as a Na^+^/K^+^-ATPase inhibitor (Pierre and Xie, 2006). Actually, the assumption is that the agonist or inhibitor function of ouabain is defined depending on which Na^+^/K^+^-ATPase population ouabain binds with: in the bulk plasma membrane, Na^+^/K^+^-ATPase is an ion pump and ouabain acts as an inhibitor; in caveolae, which are small lipid raft invaginations of the plasma membrane that function as a platform for signaling cascades, Na^+^/K^+^-ATPase interacts with several molecules, such as the structural protein caveolin-1 (Quintas et al., 2010) and the nonreceptor tyrosine kinase Src (Tian et al., 2006), and ouabain induces the activation of intracellular signals. Also, Anita Aperia’s group revealed that the N-terminus of the α-subunit of the plasma membrane Na^+^/K^+^-ATPase and of the endoplasmic reticulum IP3 receptor physically interact with each other and ouabain binding to the former may stimulate repetitive cytoplasmic Ca^2+^ transients independent on IP3 (Zhang et al., 2006). Currently, hundreds of proteins are suggested to be involved in ouabain-induced signaling (Panizza et al., 2019). As expected, this discovery opened new horizons for research on the pharmacological effects of ouabain and other cardiotonic steroids.

Different cell functions such as growth, proliferation, differentiation and membrane excitability are regulated by Ca^2+^ (Clapham, 2007), (Capiod, 2016). Calcium signaling in the CNS is a finely regulated process, as alterations in Ca^2+^ homeostasis can alter activity and induce neuronal death seen in neurodegenerative diseases and aging (Bezprozvanny, 2009), (Kumar et al., 2009). Considering the importance of Na^+^/K^+^-ATPase in brain physiology, the fact that Ca^2+^ ions are involved downstream of Na^+^/K^+^-ATPase inhibition (or activation), and that cardiotonic steroids, more specifically ouabain, may be endogenous modulators of the Na^+^/K^+^-ATPase, we conducted a systematic review of the *in vitro* effect of cardiotonic steroids on cytoplasmic Ca^2+^ in rat and mouse brain, which are the most used experimental animal models. Therefore, favoring the perception of the relationship between ouabain-Na^+^/K^+^-ATPase and homeostasis Ca^2+^ signaling in the CNS in aging and neurodegeneration.

## Materials and methods

This study followed the guidelines described in the Preferred Reporting Items for Systematic Reviews and Meta-Analyses (PRISMA), and the protocol was registered on the PROSPERO platform (Reg, No. CRD42020204498). A systematic review was performed by searching PubMed, Virtual Health Library, and EMBASE databases (between 12 June 2020 and 30 June 2020) using the following search terms: (“cardiotonic steroids” OR “cardiotonic steroid” OR “cardiac glycosides” OR “cardiac glycoside” OR digitalis OR cardenolides OR cardenolide OR bufadienolides OR bufadienolide OR ouabain OR digoxin OR digitoxin) AND (brain OR “central nervous system”) AND (calcium OR Ca^2+^). The following question guided the selection of articles and the development of this review: “What are the effects of cardiotonic steroids on calcium signaling in the central nervous system of rodents?” This question was structured according to the acronym PECO [P: Population; E: Exposure; C: Comparison; O: Outcome] and the eligibility criteria are described in Supplementary Table S1. First, articles were selected by title and abstract. No date restriction was used, however, only publications in Portuguese, English or Spanish were considered. The type of publication was also analyzed and review articles, case reports and papers presented in scientific events were excluded. Four reviewers, working in pairs, performed independent analysis, and included articles considering eligibility criteria. The third reviewer was consulted if there was no consensus on the decision.

We developed a data extraction sheet considering the following information about the included studies: author, date of publication, populations, species, gender, age, concentration of cardiotonic steroids, experimental conditions, calcium effect, and general conclusion of the articles with respect to cardiotonic steroids use. One reviewer performed data extraction from the articles that were included, and a second reviewer checked the information. Again, a third reviewer was consulted in cases of disagreement.

### Quality analysis

Most quality features and measures of risk of bias (RoB) do not apply to or are not determined for biochemical studies of the kind here reviewed, and no standard quality assessment tool exists for *in vitro* studies. We based our study on quality/RoB criteria for *in vitro* studies presented by Prueitt et al. (Prueitt et al., 2020). Questions and criteria were modified to be relevant to the evaluation of studies of calcium homeostasis/signaling. The criteria for *in vitro* studies include eight domains which have been divided into three main domains, related to outcome assessment, exposure characterization and control groups. Five other quality/RoB domains that include an analysis of the number of replicates, blinding, complete data, statistical methods, and experimental conditions were also evaluated. The studies were arranged into three levels of quality based on their classification with respect to the evaluated domains. The studies were grouped into three decreasing levels of quality, according to the eight questions addressed in the RoB analysis and identified in Table 1. Tier 1 includes studies presenting a “probably low” or “definitely low” RoB for all key domains relevant to that study type AND a “probably low” or “definitely low” RoB for most (i.e., at least half) of the other domains; Tier 2 includes studies that do not meet the criteria for Tier 1 or Tier 3. Tier 3 includes studies presenting a “probably high” or “definitely high” RoB for all key domains relevant to that study type AND a “probably high " or “definitely high” RoB for most (i.e., at least half) of the other domains. Eight quality/RoB parameters were analyzed. A study may have low RoB in some parameters, but high in others, which classifies it at an intermediate level, Tiers 2. Studies classified as Tier 3 have low quality and high RoB and those classified as Tier 1 have high quality and reliability (Prueitt et al., 2020). The evaluation of the methodological quality of the studies included in this review was done by only one author.

**TABLE 1:** Study quality/risk of bias ratings.

Study	Article	Key criteria			Other quality criteria					
		Can we be confident in the exposure characterization?	Can we be confident in the outcome assessment?	Were appropriate control groups assessed concurrently?	Did the study have an adequate number of replicates per study group?	Were experimental conditions identical across study groups?	Were research personnel blinded to test group?	Were outcome data complete?	Did the study employ appropriate statistical approaches?	Tier
Cell	Bassetti et al. (2020)	+	++	+	++	++	NR	NR	+	1
	Friess et al. (2016)	+	++	+	-	++	NR	NR	--	2
	Lomeu et al. (2003)	+	++	++	++	++	NR	NR	+	1
	Xiao et al. (2002)	+	++	++	+	++	NR	NR	+	1
	Stelmashook et al. (1999)	NR	-	+	NR	+	NR	NR	+	2
	Mark et al. (1995)	+	++	++	++	++	NR	NR	NR	1
Slices	Bai et al. (2017)	+	++	+	++	++	NR	NR	+	1
	Dietz et al. (2008)	+	++	+	--	++	NR	NR	+	2
	Basarsky et al. (1998)	NR	++	+	-	++	NR	NR	+	2
	Okamoto et al. (1994)	+	-	+	+	++	NR	NR	-	2
	Myles and Fain (1994)	NR	+	+	+	++	NR	NR	NR	2
	Mork and Geisler (1993)	+	-	+	+	+	NR	NR	NR	2
	Balduini and Costa (1990)	+	-	+	+	+	NR	NR	NR	2
	Pincus et al. (1973)	NR	-	+	+	+	NR	NR	NR	2
Synaptosomes	Satoh and Nakazato (1989)	NR	+	+	--	+	NR	NR	NR	2
	Adam-vizi and Ligeti (1986)	+	+	+	--	+	NR	NR	NR	2
	Lin et al. 1982	NR	NR	NR	NR	NR	NR	NR	NR	3
	Goddard and Robinson (1976)	NR	+	+	+	+	NR	NR	--	2
	Swanson et al. (1974)	NR	-	-	--	-	NR	NR	NR	3
	Blaustein et al. (1970)	NR	-	+	--	+	NR	NR	NR	2

++, definitely low risk of bias; +, probably low risk of bias;—or NR (not reported), probably high risk of bias; —, definitely high risk of bias.

## Results

**FIGURE 2:**
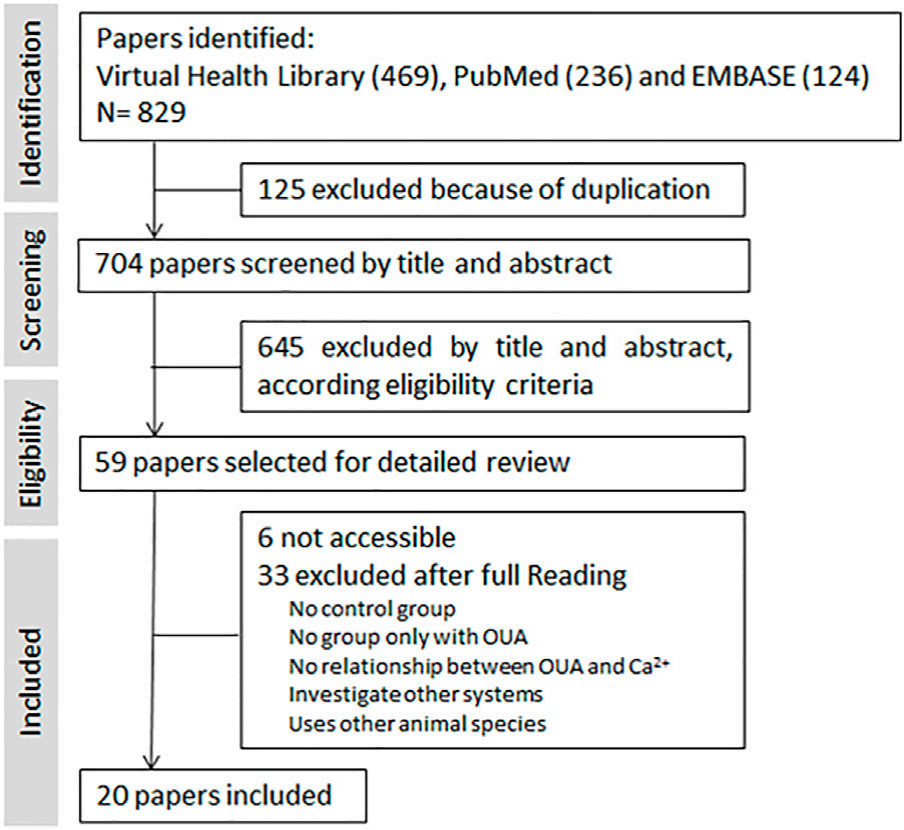
Flow diagram of study selection. The search process using the PRISMA flow diagram.

A total of 829 references were identified from electronic databases. Once duplicate entries (125) had been removed, the references were further evaluated for inclusion based on the title and/or abstract. Potentially 59 relevant articles were included in the next stage for full-text evaluation. Of these, it was not possible to gain access to 6 and 3 studies were excluded since they failed to satisfy the inclusion criteria established by the PECO. Finally, 20 studies were included in the analysis (Figure 2).

The publication period of the 20 eligible studies ranged from 1970 to 2020, and they were published in the English language. Most of the studies were conducted in the United States (*n* = 10; 50%), followed by Germany and Japan (*n* = 2; 10% each), Brazil, Canada, Denmark, and Hungary (*n* = 1; 5% each). Two trials were conducted in more than one country (10%).

Quality criteria for *in vitro* studies were applied to each article. In none of the studies experiments were blinded, and no study reported loss of data. Half of the studies did not include statistical analyses of the results. Some did not examine an adequate number of animals and poorly characterized ouabain exposure. These studies were categorized into Tier 2 or Tier 3.

Of the 20 articles, 5 (25%) were categorized into Tier 1, 13 (65%) were categorized into Tier 2, and 2 (10%) were categorized into Tier 3 based on their quality ratings across domains (Table 1). These results indicate that this systematic review presents an intermediary/low risk of bias.

Although no exclusion criteria were applied for the cardiotonic steroid studied in this systematic review, when the PECO criteria were applied, it was observed that all included studies used ouabain.

They employed different methodological strategies: six studies focused on synaptosomes, eight used brain slices, and six employed cell cultures. It was possible to group the 20 articles included in this study, on the basis of their main characteristics, which are summarized in Tables 2–4. Experimental conditions are described in Supplementary Table S2. All 20 articles are described below.

**TABLE 2:** Summary of synaptosomes studies.

Article	Population characteristics	[Ouabain]	[Ca^2+^]i levels, uptake	Major article findings in the domain of ouabain use
Satoh and Nakazato, 1989	• Brain cortex of Sprague-Dawley rats of either sex	5 × 10^−8^-5x10^−4^ M	= [Ca^2+^]i level (in absence of extracellular Ca^2+^)	Ouabain had no detectable effect on [Ca^2+^]i in the absence of extracellular Ca^2+^. However, it induced ACh release from synaptosomes, regardless of the presence or absence of extracellular Ca^2+^, which release impaired when the protein kinase C (PKC) and ryanodine receptor blocker was coincubated with ouabain
	• 220–350 g			
Adam-Vizi and Ligeti (1986)	• Rat brain cortex of CFY rats	5 × 10^−5^-5x10^−4^ M	= Ca Uptake and Efflux	Neither Ca^2+^ influx nor Ca^2+^ efflux was changed by ouabain. A slight increase of the uptake was only evoked by ouabain at a high concentration (>1 mM; data not shown)
	• 120–150 g			
	• n = 1-3 experiment made in duplicate			
Goddard and Robinson (1976)	• Rat male brains	1 × 10^−4^ M	↑122.0–185.0% Ca Uptake	Ouabain leads to an increase in uptake of^45^Ca, a high level of total calcium content and effectively prevents^45^Ca exit. The increased uptake of^45^Ca induced by ouabain was inhibited by voltage-gated sodium channels inhibitor and an inhibitor of intracellular calcium release by ryanodine receptors
	• 200–300 g		↑32.0% [Ca^2+^]i level	
	• n = 3-6		↑32.0% retention	
Swanson et al. (1974)	• Rat brain	1 × 10^−4^ M	↑33.0% Ca Uptake	OUA stimulated Ca uptake by synaptosomes
	• n = 3-4			
Blaustein and Wiesmann (1970)	• Rat brain	1 × 10^−3^ M	↑113.0% Ca Uptake	Calcium influx is increased when the internal sodium concentration is increased by treatment with ouabain
	• 200–250 g			
	• n = 3			

### Synaptosome studies

Of six papers—from 1970 to 1982—that used synaptosomes from rodent brain, five directly evaluated Ca^2+^ concentrations (Table 2). One study of the effects of ouabain in Ca^2+^ signaling observed that Ca^2+^-ATPase activity in intact synaptosomes and in brain subfractions of male ICR (Institute of Cancer Research) mice, was not affected by 1 mm ouabain (Lin and Way, 1982). Except for only one study that reported the use of forebrain, the other articles indicated the nonspecific use of rat brain.

Blaustein and Wiesmann (Blaustein and Wiesmann, 1970) and Swanson et al. (Swanson et al., 1974), observed that Ca^2+^ uptake was enhanced when 1 M × 10^−3^ M or 1 M × 10^−4^ M ouabain, respectively, was added to presynaptic synaptosomes, and this effect was directly associated with vesicles’ Na^+^ content. Goddard and Robinson (Goddard and Robinson, 1976) noted that 1 M × 10^−4^ M ouabain augmented Na^+^ uptake as well. The increased uptake of Ca^2+^ induced by ouabain was inhibited by diphenylhydantoin (DPH, well known as phenytoin), tetrodotoxin (TTX), and ruthenium red. Despite being multitarget drugs, the first two agents inhibit voltage-gated Na^+^ channels, and ruthenium red inhibits Ca^2+^ transportation, since it competes for the Ca^2+^ binding site in many proteins (Sasaki et al., 1992). On the other hand, Adam-Vizi and Ligetit, (Adam-Vizi and Ligeti, 1986), when comparing membrane depolarization and Ca^2+^ uptake, did not observe changes in Ca^2+^ uptake in brain cortical synaptosomes using 5 M × 10^−5^ − 5 M × 10^−4^ M ouabain, although membrane potential was significantly affected. Ca^2+^ influx was only detected at higher concentrations (e.g., 1 M × 10^−3^ M or more).

Moreover, acetylcholine release was not detected upon ouabain treatment, despite the degree of depolarization being comparable to those of other depolarizing agents that induce neurotransmitter exocytosis. Satoh and Nakazato (Satoh and Nakazato, 1989), showed that ouabain elicited a concentration-dependent (5 M × 10^−8^ −5 M × 10^−4^ M) release of acetylcholine from synaptosomes regardless of the presence or absence of extracellular Ca^2+^, but such effect was impaired when the protein kinase C (PKC) and ryanodine receptor blocker TMB‐8 was coincubated with ouabain, suggesting the importance of intracellular Ca^2+^.

### Brain slices studies

Of eight papers—from 1973 to 2017—that used slices from rodent brain, five directly evaluated Ca^2+^ concentrations, and all observed an increase of it (Table 3). Furthermore, three studies evaluated proteins that contribute to the control of Ca^2+^ signaling and homeostasis. These studies, using 1 M × 10^−4^ M ouabain and rat brain cortical slices, demonstrated that ouabain increased IP3 levels but had minimal effects on inositol 1,3,4,5-tetrakisphosphate (IP4) accumulation (Myles and Fain, 1994). Ouabain induced the formation of cAMP, dependently on extracellular Ca^2+^, and this process was blocked by verapamil, an inhibitor of L-type voltage-gated Ca^2+^-channels, also known as dihydropyridine receptors, that are responsible for Ca^2+^ entry during the potential action (Mørk et al., 1993). In addition, ouabain induced a concentration-dependent accumulation of inositol phosphates (InsPs), which was much higher in neonatal rat brain than in adult brain. Furthermore, the accumulation of InsPs induced by ouabain was dependent on extracellular Ca^2+^ and was blocked by EGTA (Balduini and Costa, 1990).

**TABLE 3:** Summary of brain slices studies.

Article	Population characteristics	[Ouabain]	[Ca^2+^]i level, transient or uptake	Major article findings in the domain of ouabain use
Bai et al., 2017	• Organotypic brain tissue cultures from somatosensory cortex slices	1 × 10^−3^ M	↑ 250% [Ca^2+^]i level	The [Ca^2+^]i increased and reached a maximum around 10 min after the start of ouabain perfusion and then slowly decreased while ouabain was washed out. There was also an increase in cell volume
	• Sprague-Dawley rats. Postnatal day (P) 1–2			
Dietz et al. (2008)	• Hippocampal slices (350 µm)	3 × 10^−5^ M	↑ 2000% [Ca^2+^]i level	Ouabain produced spreading depression (SD) in hippocampal slices. Before SD the Ca^2+^ signal stays near basal levels. However, after SD, large increase in Ca^2+^ signal was observed
	• Male FVB/N mice 4–6 weeks of age			
	• n = 6			
Basarsky et al. (1998)	• Hippocampal or neocortical slices (400 µm)	1 × 10^−4^ M	↑ 44 % dentate gyrus [Ca^2+^]i level	Ouabain induced SD, which started in the CA1 region, propagated across the hippocampal to the dentate gyrus. The Ca^2+^i signal increased and reached a maximum around 11 and 6 min, for dentate gyrus and astrocyte, respectively, after the start of ouabain perfusion and then decreased
	• Sprague-Dawley rats. Postnatal day (P) 15–25		↑ 40% astrocytic [Ca^2+^]i transient	
	• Dentate gyrus n = 14			
	Astrocyte n = 4			
Okamoto et al. (1994)	• Hippocampal slices (350 µm)	1 × 10^−5^ M	↑ 100% [Ca^2+^]i levels	Gradual increase in [Ca^2+^]i, which remained increased for 30 min. Treatment with lithium significantly suppressed the [Ca^2+^]i increase
	• Male Wistar rats (100–150 g)			
	• n = 4			
Pincus and Lee (1973)	• Temporal lobe slices (200 µm)	1 × 10^−4^ M	↑ 19%^45^Ca Uptake	Increased^45^Ca^2+^ uptake and dl-norepinephrine-3H (NE3H) release
	• Rats			
	• Control n = 4 Experimental n = 6			
**Article**	**Population characteristics**	**[Ouabain]**	**Ca 2+ signaling proteins**	**Major article findings in the domain of ouabain use**
Balduini and Costa (1990)	• Cerebral córtices slices (350 µm)	1 × 10^−4^ M	↑ 1271% neonatal InsPs	Ouabain induced a dose-dependent accumulation of inositol phosphates (InsPs) which was much higher in neonatal rats than in adult animals
	• Male and female Sprague Dawley rats			
	• n = 3			
Mørk et al. (1993)	• Cerebral córtices slices (330) µm	1 × 10^−4^ M	↑ 625,51% cAMP	Ouabain-induced formation of cAMP (dependent on extracellular Ca2+ and blocked by the Ca2+ channel antagonist, verapamil)
	• Male Wistar rats (180–200 g)			
	• n = 8			
Myles and Fain (1994)	• Cerebral córtices slices (350) µm	1 × 10^−4^ M	↑ 92,63% IP3	Ouabain elevates IP3 but there is little effect on IP4
	• Male Sprague Dawley rats (125–175 g)			
	• n = 9			

A large range of ouabain concentrations, from 1 × 10^−5^ to 1 × 10^−3^ M, was demonstrated to increase the [Ca^2+^]i in different rodent brain slices. Using 1 M × 10^−5^ M ouabain, Okamoto et al. (Okamoto et al., 1995) showed a progressive elevation of [Ca^2+^]i in hippocampal slices, and this was significantly suppressed with the coadministration of Li^+^, since lithium appears to antagonize the ouabain Na^+^/K^+^-ATPase inhibition, enhancing the extrusion of intracellular Ca^2+^ by Na^+^/Ca^2+^ exchanger as a consequence. Also, enhanced Ca^2+^ uptake was demonstrated using 1 M × 10^−4^ M ouabain, with a subsequent release of norepinephrine (Pincus and Lee, 1973). Ouabain at 1 M × 10^−3^ M increased the [Ca^2+^]i, reduced transmembrane water flux, and raised the mean neuron and glial cell volume (Bai et al., 2018).

In addition, concentrations of ouabain, 1 M × 10^−4^ M and 3 M × 10^−5^ M, generated *in vitro* spreading depression (SD) in freshly prepared hippocampal and neocortical slices. Interestingly, although ouabain produced a significant elevation of in [Ca^2+^ ]i, Ca^2+^ by itself was shown not to be responsible for SD (Basarsky et al., 1998), (Dietz et al., 2008).

### Cell culture studies

Of six papers—from 1995 to 2020—that used cell culture from rodent brain, all directly evaluated the Ca^2+^ concentration, and observed an increase of it (Table 4). Of the six articles, five carried out studies focused on primary cell culture. Two studies used cultures of primary brain oligodendrocyte precursor cells (OPCs), one used cortical culture, one cerebellar culture, and one hippocampal cell cultures. Only one article investigated the effects of ouabain on immortalized cells (SN56 cells). The largest increase in the Ca^2+^ level was observed at the highest ouabain concentration (1 M × 10^−3^ M) (Stelmashook et al., 1999), and the smallest increase at the lowest ouabain concentration (1 M × 10^−4^ M) (Bassetti et al., 2020). It is interesting to note that studies from the 1990s used higher concentrations of ouabain in their investigations, whereas more recent studies utilized lower concentrations. In addition to the concentration-dependent increase of the [Ca^2+]^i induced by ouabain, some articles also observed a time-dependent variation in [Ca^2+]^i (Stelmashook et al., 1999)- (Xiao et al., 2002).

**TABLE 4:** Summary of cell culture studies.

Article	Cell characteristics	[Ouabain]	[Ca^2+^]i level or transient	Major article findings in the domain of ouabain use
Bassetti et al. (2020)	• Primary brain OPCs from C57BL/6N mice	1 × 10^−7^ M	↑ 23% Ca^2+^ transients’ frequency	Increase of [Ca^2+^]i transient frequency in proximal immature OPC processes
Friess et al. (2016)	• Primary brain oligodendrocyte precursor cells (OPCs) from C57BL/6N mice	5 × 10^−7^ M	↑ 87% [Ca^2+^]i levels	Significant increase [Ca^2+^]i in OPCs and stimulated Myelin Basic Protein) synthesis
	• Postnatal day (P) 8–9			
Lomeo et al. (2003)	• SN56 cells (hybrid of septal neuronal cells from mice with the N18TG2 neuroblastoma)	2 × 10^−4^ M	↑ 250% [Ca^2+^]i level in the presence and no increase in the absence of CaCl_2_	Great increase in [Ca^2+^]i in the SN56 cholinergic cells and this increase was concentrated in the cell soma. The effect was a function of time and the maximum increase of [Ca^2+^]i in the cells was reached at 20 min. Causes a calcium-independent exocytotic release of ACh that is inhibited by blockers of intracellular calcium stores
	• n > 15			
Xiao et al. (2002)	• Primary cortical cultures	1 × 10^−4^ M	↑ 124% [Ca^2+^]i level	The [Ca^2+^]i level increased continuously, starting at ∼ 30 min after exposure, until the maximal rise in ∼90 min. This increase was largely blocked by 1M nifedipine and OUA (80 uM) exposure of 20 h induced DNA fragmentation
	• Fetal mice 15–17 d gestation			
	• n = 13-23 cells			
Stelmashook et al. (1999)	• Primary cerebellar cultures (neuro-glial) from Wistar rats	1 × 10^−3^ M	↑ 936% (20 min)	The [Ca^2+^]i level increased continuously, starting at ∼10 min after exposure, until the maximal rise in ∼35 min. The supplement of a solution with an antagonist of NMDA (1034 M, APH) together with OUA prevented cells from swelling, mitochondrial deenergization, neuronal death and increase of [Ca2+]i
	• Postnatal day (P) 7–8		↑ 2544% (35 min) [Ca^2+^]i level	
	• n = ND			
Mark et al. (1995)	• Primary hippocampal cell cultures	2 × 10^−3^ M	↑ 169% [Ca^2+^]i level	Increase in [Ca^2+^]i, which preceded neuronal degeneration
	• Embryonic rats 18 d gestation			
	• n = 9-16 cells			

ND, non-determined.

In primary cerebellar culture, Stelmashook et al. (1999) showed that addition of 1 M × 10^−3^ M ouabain had a toxic effect leading to death 62 ± 3% of the total amount of granule cells against 3 % in control. This effect was abolished when the antagonist of NMDA receptors APH (0.1 mm) was added to the incubation medium together with ouabain. APH also prevented cells from swelling, mitochondrial deenergization, neuronal death. Furthermore, in primary cortical cultures, the neuronal apoptotic and necrotic death associated with Na^+^/K^+^-ATPase inhibition, caused by the application of 1 M × 10^−4^ M ouabain, was consistent with the intracellular depletion of K^+^ and the accumulation of Ca^2+^ and Na^+^, In addition, exposure for 20 h to ouabain induced DNA fragmentation (Xiao et al., 2002). In the same way, Mark et al. (1995) using primary hippocampal cell culture using calcium indicator dye Fura-2 showed that 30 min incubation with 2 M × 10^−3^ M ouabain leads to an increase in [Ca^2+^]i levels and neuronal degeneration. They also demonstrated that the addition of ouabain promotes a decrease in neuron survival in a concentration dependent manner. Moreover, the use of Hoescht dye and ethidium bromide homodimer also revealed nuclear condensation and DNA fragmentation induced by ouabain (Mark et al., 1995).

Using SN56 cells Lomeo at al (2003) showed that ouabain (2 M × 10^−4^ M) had a calcium dependent effect on [Ca^2+^]i levels, leading to enhanced acetylcholine release. This effect of ouabain on acetylcholine release was dose and time dependent, achieving the maximum value after 30 min and was not inhibited by the addition of 1 µM tetrodotoxin (TTX). However, the effect of ouabain was suppressed with the addition of BAPTA-AM (Lomeo et al., 2003).

Interestingly, studies using cultures of oligodendrocyte precursor cells have shown that a long incubation of oligodendrocyte precursor cells (OPC) cultures with ouabain (5 M × 10^−7^ M, 24 h) failed to significantly change [Na^+^]i levels, but ouabain treatment significantly increased [Ca^2+^]i and stimulated myelin basic protein synthesis (Friess et al., 2016).

**FIGURE 3:**
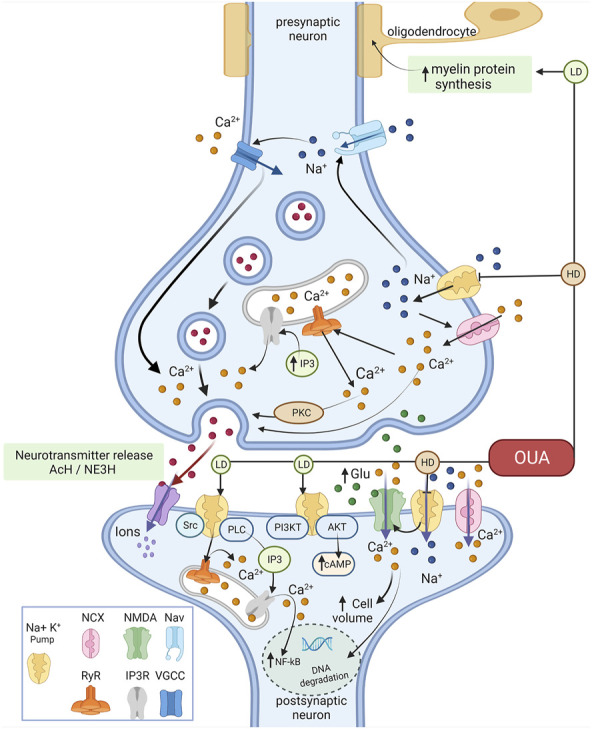
Representative schedule of the effects of ouabain on Ca^2+^ signaling. Ouabain in high concentrations selectively binds to Na^+^/K^+^-ATPase, inhibiting its pump activity, leading to increased intracellular Na^+^ and Ca^2+^ concentrations. The latter by inducing a lower/reverse action of the Na^+^–Ca^2+^ exchanger (NCX) colocalizes with the Na^+^/K^+^-ATPase. Ouabain effects on intracellular Ca^2+^ influence the release of acetylcholine (Ach) and norepinephrine (NE3H) and glutamate (Glu). Binding of ouabain to Na^+^/K^+^-ATPase triggers intracellular signaling networks in the glutamate signaling cascade through protein-protein interactions, generating many effects independent of the impairment of electrochemical gradients. In this case, ouabain (and other cardiotonic steroids) would act as an agonist, stimulating these pathways, acting in a different way observed as Na^+^/K^+^-ATPase inhibitor. In low concentrations ouabain can stimulate changes in Na^+^/K^+^-ATPase that triggers signaling complexes such PI3K-AKT pathways, increasing cAMP. Also, ouabain modulates Ca^2+^ intracellular oscillation through activation of ryanodine receptor (RyR) and IP3 receptor (IP3R) that provoke the increasing of NF-κB and activation of PKC. LD- Low Dose and HD- High dose (Figure were created with BioRender.com).

Figure 3 is a schematic summary of the finding of this study.

## Discussion

The most studied effects of cardiotonic steroids refer to their action on the cardiovascular system. Here in this systematic review, we demonstrated the effects of ouabain on Ca^2+^ oscillation and signaling in the nervous system of rodents, indicating that the balance between ouabain-induced neuroprotective and neurotoxic effects is concentration-dependent. Furthermore, the action of ouabain is broad, acting not only on neurons but also on glial cells.

Ca^2+^ homeostasis plays a crucial role in the maintenance of different cellular functions. Ca^2+^ has been described as an important second messenger, regulating many different cellular processes, including cell division, proliferation differentiation, apoptosis, necrosis, neurotransmission and synaptic plasticity (Arundine and Tymianski, 2003), (Berridge et al., 2003). The concentration of this free ion in the cytosol is kept about 10,000 times below the extracellular concentration (Nicotera and Orrenius, 1998). This high electrochemical Ca^2+^ gradient between the intra and extracellular compartments enables the transduction of biochemical signals into cells (Berridge et al., 2003).

Regarding neurotransmission modulated by ouabain, Ca^2+^ triggers synaptic vesicle exocytosis, thereby releasing the neurotransmitters contained in the vesicles and initiating synaptic transmission (Katz and Miledi, 1967). Whereas ouabain selectively binds to Na^+^/K^+^-ATPase and inhibits its ion pump activity, increasing intracellular Na^+^ and Ca^2+^ concentrations, the latter by inducing a lower/reverse activity of the Na^+^/Ca^2+^ exchanger that colocalizes with the Na^+^/K^+^-ATPase (Larsen et al., 2016), (Kinoshita et al., 2020), it is expected that it can modulate neurotransmission. Inhibition of the Na^+^/K^+^-ATPase could lead to depolarization of the neuron, followed by Ca^2+^ influx and transmitter release by exocytosis (Banks, 1967). It could also lead indirectly to a rise in intracellular Ca^2+^, through mobilization of intracellular Ca^2+^ stores (Baker and Crawford, 1975). The results collected in this study represents the literature. In fact, ouabain is capable of interfering with different neurotransmission systems, such as cholinergic (Satoh and Nakazato, 1989), (v Blasi et al., 1988), noradrenergic (Pincus and Lee, 1973), (Yamazaki et al., 2007) and glutamatergic (Jacobson et al., 1986), (Stelmashook et al., 1999). Moreover, other studies have demonstrated that ouabain also interferes with the release of dopamine in animal models (Sui et al., 1199).

The toxic effect of high concentrations of ouabain has been widely described in the literature, as well as the involvement of high levels of Ca^2+^ influx, promoting neuronal excitotoxicity (Veldhuis et al., 2003). This effect was observed in synaptosomes as well as in brain slices.

Excitotoxicity is a term used to indicate the death of nerve cells by glutamate (Glu) as well as other amino acids, resulting in neurodegenerative diseases (Lewerenz and Maher, 2015), (Olney, 1986), increased release of Glu that occurs under neurological disorders may be a result of metabolic changes and reduced Na^+^,K^+^-ATPase activity (Shi et al., 2019), (Beal et al., 1993). The findings indicate that NMDA receptors are involved in ouabain effects on [Ca^2+^]i and cell toxicity. It is known that Na^+^/K^+^-ATPase inhibition leads to a decrease in Glu uptake in cortical astrocytes cell cultures (Volterra et al., 1994), interfering with GluT transport in astrocytes (Nguyen et al., 2010), favoring the neurotoxic effects of high doses of ouabain. This suggesting that Na^+^/K^+^-ATPase inhibition by ouabain led to Glu accumulation of extracellular Glu, hyperstimulation of glutamate receptors, and higher Ca^2+^ and Na^+^ influxes into the cells through N-methyl-d-aspartate (NMDA) receptors in neuro–glial cell cultures of the cerebellum (Stelmashook et al., 1999). These authors associated the exposure of high ouabain concentrations with a toxic effect on cerebellar and hippocampal cells.

Interestingly, ouabain in nanomolar concentration consistently reduces the Ca^2+^ response to NMDA. Downregulation of the NMDA response is not associated with internalization of the receptor or with alterations in its state of Src phosphorylation (Akkuratov et al., 2020). It has been observed that ouabain activates NF-κB by an NMDA–Src–Ras-like protein through MAPK pathways in cultured cerebellar cells (de Sá Lima et al., 2013). In addition, the intra-hippocampal administration of ouabain in a low concentration that did not alter the activity of Na^+^/K^+^-ATPase promoted the activation of NF-κB, leading to increased brain-derived neurotrophic factor (BDNF) levels, similar to NMDA treatment, which was reversed by the NMDA antagonist MK-801 (Kawamoto et al., 2012b). Moreover, intrahippocampal injection of ouabain 10 nM activated the Wnt/β-catenin signaling pathway and to increase CREB/BDNF and NFκB levels. These effects contribute to important changes in the cellular microenvironment, resulting in enhanced levels of dendritic branching in hippocampal neurons, in association with an improvement in spatial reference memory and the inhibition of long-term memory extinction (Orellana et al., 2018).

Altered levels of acetylcholine, as well as its receptors, have been observed in neurodegenerative and neuropsychiatric diseases (Tata et al., 2014). In order to obtain a concentration-dependent curve of [^3^H] acetylcholine release, Satoh and Nakazato (Satoh and Nakazato, 1989) utilized 5 × 10^−8^-5x10^−4^ M ouabain, possibly inhibiting initially the α_2_/α_3_ isoforms—which are, in rodents, much more sensitive to cardiotonic steroids—and subsequently the ouabain-resistant α_1_ (O’brien et al., 1994), (Lopez et al., 2002). Interestingly Lomeo at al (2003) using SN56 cells showed that ouabain had a Ca^2+^ dependent effect on [Ca^2+^]i levels, leading to enhanced acetylcholine release. This effect of ouabain on acetylcholine release was dose and time dependent, achieving the maximum value after 30 min. This effect was not inhibited by the addition of 1 µM tetrodotoxin (TTX), discarding the involvement of TTX-sensitive Na^+^ channels. However, the effect of ouabain was suppressed with the addition of BAPTA-AM, suggesting the involvement of intracellular calcium stores. The authors suggested that in cholinergic neurons the ouabain induced increase in [Na^+^]_i_ results in intracellular calcium alterations inducing an increase in [Ca^2+^]_i_, causing a release of acetylcholine independent of Ca^2+^ (Lomeo et al., 2003). Despite the effect of high doses of ouabain on cholinergic neurotransmission, no studies were found on the role of lower doses of this cardiotonic on the cholinergic system.

Another phenomenon observed on the effect of the micromolar application of ouabain was the Spreading depression (SD), which is a wave of profound depolarization that propagates throughout the brain tissue after traumatic or vascular brain insults (Somjen, 2001). Interestingly, it was observed that the spreading depression is not only caused by the increase of in [Ca^2+^ ]i, but also the partition of Zn^2+^ and mitochondrial stress, since it was observed that selective chelation of Zn^2+^ with N,N,N,N-tetrakis (2-pyridylmethyl) ethylenediamine (TPEN) eliminated ouabain-SD, implying that Zn^2+^ entry and mitochondrial dysfunction may play a critical role in the Ouabain-induced SD mechanism (Dietz et al., 2008). Thus, this is a useful model for studying the pathways involved in this phenomenon and that can lead to the search for more efficient treatments.

Different studies have demonstrated that neuronal activity promotes an elevation in extracellular [K^+^], stimulating an increase in intracellular Ca^2+^transient in oligodendrocytes, due to the reversion of the Na^+^/Ca^2+^ exchanger, which led to increased synthesis of myelin basic protein (MBP) (Belachew et al., 2000), (Chen et al., 2007). Interestingly, using primary brain OPCs cultures, Friess et al. (Friess et al., 2016) showed that incubation with 5 M × 10^−7^ M ouabain increased the intracellular Ca^2+^ levels and stimulated MBP synthesis, and this effect of ouabain on Ca^2+^ transients was eliminated by the addition to the incubation medium of 1 µMKB-R734 9, a Na^+^/Ca^2+^ exchanger inhibitor. In addition, it was observed that the activity of the Na^+^/K^+^-ATPase α_2_ isoform, present in oligodendrocyte lineage cell (OLCs), changed the Na^+^/Ca^2+^ exchanger-mediated [Ca^2+^]i, modulating MBP synthesis in OLCs (Hammann et al., 2018). Using the same experimental model of primary OPCs cultures, Basseti et al. (Bassetti et al., 2020) demonstrated that lower concentrations of ouabain (1 M × 10^−7^ M) also increased Ca^2+^ transient frequency. Additionally, this work showed that the inhibition of ryanodine receptor type 3 with 10 µM ryanodine also blocked Ca^2+^ transient.

The process of myelination of neuronal axons through oligodendrocyte activity is a process controlled by the release of neurotransmitters and changes in ionic concentrations (Butt and Bay, 2011). Among the important proteins in the myelination process, there is myelin basic protein (MBP), and failures in its production result in CNS hypomyelination processes (Readhead and Hood, 1990), (Wiecien et al., 1998). Interestingly, studies using cultures of oligodendrocyte precursor cells (OPC) have shown that a long incubation (24 h) of these cells with nanomolar concentrations of ouabain failed to significantly change [Na^+^]i levels, but ouabain treatment significantly increased [Ca^2+^]i and stimulated MPB synthesis (Friess et al., 2016). The same group further suggested that the crosstalk among ryanodine receptors, Na^+^/–Ca^2+^ exchangers, and possibly Na^+^/K^+^-ATPase may evoke Ca^2+^ transients for the development of isolated oligodendrocytes (Bassetti et al., 2020), thus demonstrating the importance of Na^+^,K^+^-ATPase in the synthesis of axonal myelin by oligodendrocytes.

Interestingly, in addition to the existence of a Na^+^/Ca^2+^ exchanger colocalized with Na^+^/K^+^-ATPase, the discovery of ancillary signaling pathways, involving protein–protein interactions, revealed that the physical association between Na^+^/K^+^-ATPase and IP3 receptors allows intracellular Ca^2+^ oscillations evoked by ouabain (Fontana et al., 2013), (Miyakawa-Naito et al., 2003). Moreover, Src kinase activation would promote the stimulation of phospholipase C and PKC independently of ion modulation (Mohammadi et al., 2001), (Yuan et al., 2005).

One study in brain slices demonstrated that ouabain caused the stimulation of PtdIns hydrolysis, causing an accumulation of InsPs in the cytoplasm, which may be related to cell signaling modulated by Na^+^/K^+^-ATPase inhibition, activation of Na^+^/Ca^2+^ exchanger, mobilization of Ca^2+^ and PKC (Balduini and Costa, 1990). Interestingly, there was no direct correlation between the stimulus leading to PtdIns hydrolysis and the binding of radioactive ouabain (number of Na^+^/K^+^-pumps) or strophantidin effect (an equipotent inhibitor), and this may suggest that this outcome is modulated by cell signaling mechanisms besides the classic inhibitory effect. A similar effect was found in the study by Myles et al. (Myles and Fain, 1994), where 1 M × 10^−4^ M ouabain stimulated an 82% increase in intracellular IP3 levels but had a minimal effect on IP4 accumulation. Moreover, in addition to InsPs, treatment with 1 M × 10^−4^ M ouabain in brain slices also increased intracellular cAMP (Mørk et al., 1993). Since only few studies utilized slices, it is not possible to say whether accumulation of cytosolic Ca^2+^ found in these studies is an effect modulated only by the regulation of the Na^+^/Ca^2+^ exchanger, which is the classic effect of cardiotonic steroids. It is well known that Ca^2+^ mobilization and influx is linked to the activation of cellular signaling pathways, especially those associated with G proteins, with the involvement of cAMP and IP3 (Satoh and Nakazato, 1989), (Liu et al., 2007).

In addition, studies that include the nervous system and others demonstrate that Na^+^/K^+^-ATPase acts as a receptor and a signal transducer, involving many pathways such as that dependent on the membrane-associated nonreceptor tyrosine kinase Src (Tian et al., 2006) the Ras/Raf/ERK1/2 pathway (Eckert et al., 2008), the phosphate inositol 3-kinase (PI3K) pathway, the PI3K-dependent protein kinase B pathway, phospholipase C signaling, [Ca^2+^]i oscillations (Liu et al., 2007), (Schoner and Scheiner-Bobis, 2007), (Aperia et al., 2015), and gene transcription (Li et al., 2006). These pathways are also triggered by the interaction of ouabain with Na^+^/K^+^-ATPase. Na^+^/K^+^- ATPase inhibition or activation is dependent on ouabain concentration. It has been shown that inhibition of pump activity requires micromolar concentrations of ouabain (Fontana et al., 2013), (Blaustein and Hamlyn, 2020), while activation of Na^+^/K^+^-ATPase signaling pathways occurs in the presence of nanomolar concentrations of ouabain (Fontana et al., 2013).

As discussed above, several studies using different models have shown that the effects of ouabain at low concentrations are due to the activation of cell signaling pathways. Studies have shown that in renal epithelial cells, as well as in astrocytes, ouabain, at low concentrations, binds with Na^+^/K^+^-ATPase directly activating the IP3 receptors (physical interaction), and triggering slow Ca^2+^ oscillations and the activation of NF-kB; this ultimately leads to the proliferation of these cells (Liu et al., 2007), (Li et al., 2006), (Aizman et al., 2001). Furthermore, studies in cardiomyocytes have observed that low ouabain concentrations promote [Ca^2+^]i oscillations due to the activation of Src kinase followed by the stimulation of the Ras/Raf/MEK/MAPK cascade regulating cell hypertrophic growth (Yuan et al., 2005), (Zhu et al., 1996). It is important to note that in such signaling conditions, Ca^2+^ is a key factor, and positive feedback may occur.

The therapeutic range for the use of ouabain and other cardiac steroids is very narrow. Added to this problem of narrow therapeutic index, we have the majority use of this drug by elderly patients, contributing to toxicity problems (Whayne, 12018). This reality caused mistrust and disuse of cardiac steroids in clinical practice. Thus, in addition to expanding knowledge about signaling pathways and neuroprotective effects of low doses of ouabain and other cardiotonic, a strategy for the safe therapeutic use of cardiotonic steroids for neuroprotection would be the chemical modification of their structure to increase the therapeutic index of this class. Gamma-benzylidene digoxin derivatives are digoxin-derived molecules that have demonstrated low toxicity to cells (de Oliveira et al., 2021) and have already demonstrated neuroprotection for chemical ischemia (de Souza Gonçalves et al., 2019) and increased α3 activity and increased antioxidant defenses such as GSH, desired drug characteristics of neuroprotection (Parreira et al., 2021).

## Conclusion

Ca^2+^ mobilization is a canonical effect of cardiotonic steroids such as ouabain. In all models studied - synaptosomes, brain slices or cell cultures—an increase in [Ca^2+^]i was observed. In addition to the well known cytotoxic effects of ouabain, resulting from stimulation of the Na^+^/Ca^2+^ exchanger reverse mode and increased Ca^2+^, other effects have been reported, since Ca^2+^ may play a role in major cellular effects, mainly by activating signaling pathways. Ouabain-induced Ca^2+^ signaling was able to increment cholinergic, noradrenergic and glutamatergic neurotransmission. Treatment with ouabain stimulated MBP synthesis and significantly increased biological second messengers such as InsPs, IP3 and cAMP. This review deepens the knowledge about the effects and signaling mediated by cardiotonic steroids (ouabain) in the nervous system, which has been shown to be concentration dependent. Structural modifications of cardiotonic steroids may be useful for the generation of new agents that are less toxic and with neuroprotective action.

## Data Availability

The raw data supporting the conclusions of this article will be made available by the authors, without undue reservation.

## References

[B1] Adam-Vizi V. Ligeti E. (1986). Calcium uptake of rat brain synaptosomes as a function of membrane potential under different depolarizing conditions. J. Physiol. 372 (1), 363–377. 10.1113/jphysiol.1986.sp016013 3723411 PMC1192767

[B2] Aizman O. Uhlén P. Lal M. Brismar H. Aperia A. (2001). Ouabain, a steroid hormone that signals with slow calcium oscillations. Proc. Natl. Acad. Sci. U. S. A. 98 (23), 13420–13424. 10.1073/pnas.221315298 11687608 PMC60886

[B3] Akera T. Brody T. M. (1977). The role of Na+, K+-ATPase in the inotropic action of digitalis. Pharmacol. Rev. 29 (3), 187–220. 150607

[B4] Akkuratov E. E. Westin L. Vazquez-Juarez E. de Marothy M. Melnikova A. K. Blom H. (40302020). Ouabain modulates the functional interaction between Na, K-ATPase and NMDA receptor. Mol. Neurobiol. 57 (10), 4018–4030. 10.1007/s12035-020-01984-5 32651756 PMC7467916

[B5] Aperia A. Akkuratov E. E. Fontana J. M. Brismar H. (2015). Hugh davson distinguished lectureship of the cell and molecular physiology sectionNa+-k+-ATPase, a new class of plasma membrane receptors. Am. J. Physiol. Cell Physiol. 310 (7), C491–C495. 10.1152/ajpcell.00359.2015 26791490

[B6] Arundine M. Tymianski M. (2003). Molecular mechanisms of calcium-dependent neurodegeneration in excitotoxicity. Cell Calcium 34 (4–5), 325–337. 10.1016/S0143-4160(03)00141-6 12909079

[B7] Bai R. Springer C. S. Plenz D. Basser P. J. (2018). Fast, Na+/K+ pump driven, steady-state transcytolemmal water exchange in neuronal tissue: A study of rat brain cortical cultures. Magn. Reson. Med. 79 (6), 3207–3217. 10.1002/mrm.26980 29106751

[B8] Baker P. F. Crawford A. C. (1975). A note of the mechanism by which inhibitors of the sodium pump accelerate spontaneous release of transmitter from motor nerve terminals. J. Physiol. 247 (1), 209–226. 10.1113/jphysiol.1975.sp010928 166163 PMC1309462

[B9] Balduini W. Costa L. G. (1990). Characterization of ouabain-induced phosphoinositide hydrolysis in brain slices of the neonatal rat. Neurochem. Res. 15 (10), 1023–1029. 10.1007/BF00965749 1963925

[B10] Banks P. (1967). The effect of ouabain on the secretion of catecholamines and on the intracellular concentration of potassium. J. Physiol. 193 (3), 631–637. 10.1113/jphysiol.1967.sp008383 16992301 PMC1365518

[B11] Basarsky T. A. Duffy S. N. Andrew R. D. MacVicar B. A. (1998). Imaging spreading depression and associated intracellular calcium waves in brain slices. J. Neurosci. 18 (18), 7189–7199. 10.1523/jneurosci.18-18-07189.1998 9736642 PMC6793239

[B12] Bassetti D. Hammann J. Luhmann H. J. White R. Kirischuk S. (2020). Ryanodine receptor- and sodium-calcium exchanger-mediated spontaneous calcium activity in immature oligodendrocytes in cultures. Neurosci. Lett. 732. 10.1016/j.neulet.2020.134913 32482568

[B13] Beal M. F. Hyman B. T. Koroshetz W. (1993). Do defects in mitochondrial energy metabolism underlie the pathology of neurodegenerative diseases? Trends Neurosci. 16 (4), 125–131. 10.1016/0166-2236(93)90117-5 7682343

[B14] Belachew S. Malgrange B. Rigo J. M. Rogister B. LePrince P. Hans G. (2000). Glycine triggers an intracellular calcium influx in oligodendrocyte progenitor cells which is mediated by the activation of both the ionotropic glycine receptor and Na+-dependent transporters. Eur. J. Neurosci. 12 (6), 1924–1930. 10.1046/j.1460-9568.2000.00085.x 10886333

[B15] Berridge M. J. Bootman M. D. Roderick H. L. (2003). Calcium signalling: Dynamics, homeostasis and remodelling. Nat. Rev. Mol. Cell Biol. 4 (7), 517–529. 10.1038/nrm1155 12838335

[B16] Bezprozvanny I. (2009). Calcium signaling and neurodegenerative diseases. Trends Mol. Med. 15 (3), 89–100. 10.1016/j.molmed.2009.01.001 19230774 PMC3226745

[B17] Blanco G. Mercer R. W. (1998). Isozymes of the Na-K-ATPase: Heterogeneity in structure, diversity in function. The American Physiological Society, 245–247. 10.1177/153857449803200307 9815123

[B18] Blanco G. (2005). Na, K-ATPase subunit heterogeneity as a mechanism for tissue-specific ion regulation. Semin. Nephrol. 25 (5), 292–303. 10.1016/j.semnephrol.2005.03.004 16139684

[B19] Blaustein M. P. Hamlyn J. M. (2020). Ouabain, endogenous ouabain and ouabain-like factors: The Na+ pump/ouabain receptor, its linkage to NCX, and its myriad functions. Cell Calcium 86, 102159. 10.1016/j.ceca.2020.102159 31986323

[B20] Blaustein M. P. Juhaszova M. Golovina V. A. Church P. J. Stanley E. F. (2002). Na/Ca exchanger and PMCA localization in neurons and astrocytes: Functional implications. Ann. N. Y. Acad. Sci. 976, 356–366. 10.1111/j.1749-6632.2002.tb04762.x 12502582

[B21] Blaustein M. P. Wiesmann W. P. (1970). Effect of sodium ions on calcium movements in isolated synaptic terminals. Proc. Natl. Acad. Sci. U. S. A. 66 (3), 664–671. 10.1073/pnas.66.3.664 5269232 PMC283102

[B22] Brini M. Calì T. Ottolini D. Carafoli E. (2014). Neuronal calcium signaling: Function and dysfunction. Cell. Mol. Life Sci. 71 (15), 2787–2814. 10.1007/s00018-013-1550-7 24442513 PMC11113927

[B23] Butt A. M. Bay V. (2011). Axon-glial interactions in the central nervous system. J. Anat. 219 (1), 1. 10.1111/j.1469-7580.2011.01401.x 21644971 PMC3130154

[B24] Capiod T. (2016). Extracellular calcium has multiple targets to control cell proliferation. Adv. Exp. Med. Biol. 898, 133–156. 10.1007/978-3-319-26974-0_7 27161228

[B25] Carvalho D. C. M. Cavalcante-Silva L. H. A. Lima E. d. A. Galvao J. G. F. M. Alves A. K. d. A. Feijo P. R. O. (2019). Marinobufagenin inhibits neutrophil migration and proinflammatory cytokines. J. Immunol. Res. 2019, 1094520. 10.1155/2019/1094520 31236418 PMC6545758

[B26] Cereijido M. Contreras R. G. Shoshani L. Larre I. (2012). The Na +-K +-ATPase as self-adhesion molecule and hormone receptor. Am. J. Physiol. Cell Physiol. 302 (3), C473–C481. 10.1152/ajpcell.00083.2011 22049208

[B27] Chen H. Kintner D. B. Jones M. Matsuda T. Baba A. Kiedrowski L. (2007). AMPA-mediated excitotoxicity in oligodendrocytes: Role for Na +-K+-Cl- co-transport and reversal of Na +/Ca2+ exchanger. J. Neurochem. 102 (6), 1783–1795. 10.1111/j.1471-4159.2007.04638.x 17490438

[B28] Clapham D. E. (2007). Calcium signaling. Cell 131 (6), 1047–1058. 10.1016/j.cell.2007.11.028 18083096

[B29] Clausen M. v. Hilbers F. Poulsen H. (2017). The structure and function of the Na, K-ATPase isoforms in health and disease. Front. Physiol. 8 (JUN), 371–416. 10.3389/fphys.2017.00371 28634454 PMC5459889

[B30] de Oliveira G. C. Rocha S. C. da Silva Lopes M. A. Paixão N. Alves S. L. G. Pessoa M. T. C. (2021). Implications of synthetic modifications of the cardiotonic steroid lactone ring on cytotoxicity. J. Membr. Biol. 254 (5–6), 487–497. 10.1007/s00232-021-00186-x 34128090

[B31] de Sá Lima L. Kawamoto E. M. Munhoz C. D. Kinoshita P. F. Orellana A. M. Curi R. (2013). Ouabain activates NFκB through an NMDA signaling pathway in cultured cerebellar cells. Neuropharmacology 73, 327–336. 10.1016/j.neuropharm.2013.06.006 23774137

[B32] de Souza Gonçalves B. de Moura Valadares J. M. Alves S. L. G. Silva S. C. Rangel L. P. Cortes V. F. (2019). Evaluation of neuroprotective activity of digoxin and semisynthetic derivatives against partial chemical ischemia. J. Cell. Biochem. 120 (10), 17108–17122. 10.1002/jcb.28971 31310381

[B33] Dietz R. M. Weiss J. H. Shuttleworth C. W. (2008). Zn2+ influx is critical for some forms of spreading depression in brain slices. J. Neurosci. 28 (32), 8014–8024. 10.1523/JNEUROSCI.0765-08.2008 18685026 PMC2577031

[B34] Dobretsov M. Stimers J. R. (2005). Neuronal function and alpha3 isoform of the Na/K-ATPase. Front. Biosci. 10 (1–3), 2373–2396. 10.2741/1704 15970502

[B35] Eckert A. Tagscherer K. E. Haas T. L. Grund K. Sykora J. (2008). The PEA-15/PED protein protects glioblastoma cells from glucose deprivation-induced apoptosis via the ERK/MAP kinase pathway. Oncogene 27 (8), 1155–1166. 10.1038/sj.onc.1210732 17700518

[B36] Fedorova O. v. Bagrov A. Y. (1997). Inhibition of Na/K ATPase from rat aorta by two Na/K pump inhibitors, ouabain and marinobufagenin: Evidence of interaction with different α- subunit isoforms. Am. J. Hypertens. 10 (8), 929–935. 10.1016/S0895-7061(97)00096-4 9270089

[B37] Fedorova O. v. Kolodkin N. I. Agalakova N. I. Lakatta E. G. Bagrov A. Y. (2001). Marinobufagenin, an endogenous alpha-1 sodium pump ligand, in hypertensive Dahl salt-sensitive rats. Hypertension 37 (2 II), 462–466. 10.1161/01.hyp.37.2.462 11230319

[B38] Fontana J. M. Burlaka I. Khodus G. Brismar H. Aperia A. (2013). Calcium oscillations triggered by cardiotonic steroids. FEBS J. 280 (21), 5450–5455. 10.1111/febs.12448 23890276

[B39] Friess M. Hammann J. Unichenko P. Luhmann H. J. White R. Kirischuk S. (2016). Intracellular ion signaling influences myelin basic protein synthesis in oligodendrocyte precursor cells. Cell Calcium 60 (5), 322–330. 10.1016/j.ceca.2016.06.009 27417499

[B40] Geering K. (2008). Functional roles of Na, K-ATPase subunits. Curr. Opin. Nephrol. Hypertens. 17 (5), 526–532. 10.1097/MNH.0b013e3283036cbf 18695395

[B41] Goddard G. A. Robinson J. D. (1976). Uptake and release of calcium by rat brain synaptosomes. Brain Res. 110 (2), 331–350. 10.1016/0006-8993(76)90406-6 938947

[B42] Godinho A. N. Costa G. T. Oliveira N. O. Cardi B. A. Uchoa D. E. A. Silveira E. R. (2017). Effects of cardiotonic steroids on isolated perfused kidney and NHE3 activity in renal proximal tubules. Biochim. Biophys. Acta. Gen. Subj. 1861 (8), 1943–1950. 10.1016/j.bbagen.2017.05.012 28506883

[B43] Hammann J. Bassetti D. White R. Luhmann H. J. Kirischuk S. (2018). α2 isoform of Na + , K + -ATPase via Na + , Ca 2+ exchanger modulates myelin basic protein synthesis in oligodendrocyte lineage cells *in vitro* . Cell Calcium 73, 1–10. 10.1016/j.ceca.2018.03.003 29880193

[B44] Jacobson I. Hagberg H. Sandberg M. Hamberger A. (1986). Ouabain-induced changes in extracellular aspartate, glutamate and GABA levels in the rabbit olfactory bulb *in vivo* . Neurosci. Lett. 64 (2), 211–215. 10.1016/0304-3940(86)90102-3 2870447

[B45] Katz B. Miledi R. (1967). Ionic requirements of synaptic transmitter release. Nature 215 (5101), 651. 10.1038/215651a0 4292912

[B46] Kawamoto E. M. Lima L. S. Munhoz C. D. Yshii L. M. Kinoshita P. F. Amara F. G. (2012). Influence of N-methyl-D-aspartate receptors on ouabain activation of nuclear factor-κB in the rat hippocampus. J. Neurosci. Res. 90 (1), 213–228. 10.1002/jnr.22745 22006678

[B47] Kawamoto S. Tran T. H. Maruya M. Suzuki K. Doi Y. Tsutsui Y. (2012). The inhibitory receptor PD-1 regulates IgA selection and bacterial composition in the gut. Science 821, 485–489. 10.1126/science.1217718 22539724

[B48] Kinoshita P. F. Orellana A. M. M. Nakao V. W. de Souza Port's N. M. Quintas L. E. M. Kawamoto E. M. (2020). The Janus face of ouabain in Na+/K+-ATPase and calcium signalling in neurons. Br. J. Pharmacol. 179, 1512–1524. 10.1111/bph.15419 33644859

[B49] Koh C. H. Wu J. Chung Y. Y. Liu Z. Zhang R. R. Chong K. (2017). Identification of Na+/K+-ATPase inhibition-independent proarrhythmic ionic mechanisms of cardiac glycosides. Sci. Rep. 7 (1), 2465. 10.1038/s41598-017-02496-4 28550304 PMC5446409

[B50] Kumar A. Bodhinathan K. Foster T. C. (2009). Susceptibility to calcium dysregulation during brain aging. Front. Aging Neurosci. 1 (NOV), 2–13. 10.3389/neuro.24.002.2009 20552053 PMC2874411

[B51] Kupicha F. K. (1982). Studies on african apocynaceae: The genus Acokanthera. Kew Bull. 37 (1), 41. 10.2307/4114719

[B52] Larsen B. R. Stoica A. MacAulay N. (2016). Managing brain extracellular K+ during neuronal activity: The physiological role of the Na+/K+-ATPase subunit isoforms. Front. Physiol. 7 (APR), 141–210. 10.3389/fphys.2016.00141 27148079 PMC4841311

[B53] Lees G. J. Lehmann A. Sandberg M. Hamberger A. (1990). The neurotoxicity of ouabain, a sodium-potassium ATPase inhibitor, in the rat hippocampus. Neurosci. Lett. 120 (2), 159–162. 10.1016/0304-3940(90)90027-7 1705675

[B54] Lewerenz J. Maher P. (2015). Chronic glutamate toxicity in neurodegenerative diseases-What is the evidence? Front. Neurosci. 9 (DEC), 469–520. 10.3389/fnins.2015.00469 26733784 PMC4679930

[B55] Li J. Zelenin S. Aperia A. Aizman O. (2006). Low doses of ouabain protect from serum deprivation-triggered apoptosis and stimulate kidney cell proliferation via activation of NF-kappaB. J. Am. Soc. Nephrol. 17 (7), 1848–1857. 10.1681/ASN.2005080894 16707566

[B56] Lin S. C. Way E. L. (1982). Calcium‐activated ATPases in presynaptic nerve endings. J. Neurochem. 39 (6), 1641–1651. 10.1111/j.1471-4159.1982.tb07998.x 6216324

[B57] Lingrel J. B. (2009). The physiological significance of the cardiotonic steroid/ouabain-binding site of the Na, K-ATPase. Annu. Rev. Physiol. 72, 395–412. 10.1146/annurev-physiol-021909-135725 PMC3079441 20148682

[B58] Liu X. L. Miyakawa A. Aperia A. Krieger P. (2007). Na, K-ATPase generates calcium oscillations in hippocampal astrocytes. NeuroReport 18 (6), 597–600. 10.1097/WNR.0b013e3280b07bc9 17413664

[B59] Lomeo R. S. Gomez R. S. Prado M. A. M. Romano-Silva M. A. Massensini A. R. Gomez M. v. (2003). Exocytotic release of [3H]-Acetylcholine by ouabain involves intracellular Ca2+ stores in rat brain cortical slices. Cell. Mol. Neurobiol. 23 (6), 917–927. 10.1023/B:CEMN.0000005320.06215.80 14964779 PMC11530174

[B60] Lopez L. B. Eduardo Quintas L. M. Noel F. U. (2002). Influence of development on Na(+)/K(+)-ATPase expression: Isoform- and tissue-dependency. Comp. Biochem. Physiol. A Mol. Integr. Physiol. 131 (2), 323–333. 10.1016/s1095-6433(01)00482-2 11818222

[B61] Mark R. J. Hensley K. Butterfield D. A. Mattson M. P. (1995). Amyloid β-peptide impairs ion-motive ATPase activities: Evidence for a role in loss of neuronal Ca2+ homeostasis and cell death. J. Neurosci. 15 (9), 6239–6249. 10.1523/jneurosci.15-09-06239.1995 7666206 PMC6577674

[B62] McGarry S. J. Williams A. J. (1993). Digoxin activates sarcoplasmic reticulum Ca2+‐release channels: A possible role in cardiac inotropy. Br. J. Pharmacol. 108 (4), 1043–1050. 10.1111/j.1476-5381.1993.tb13503.x 8387382 PMC1908139

[B63] Miyakawa-Naito A. Uhlen P. Lal M. Aizman O. Mikoshiba K. Brismar H. (2003). Cell signaling microdomain with Na, K-ATPase and inositol 1, 4, 5-trisphosphate receptor generates calcium oscillations. J. Biol. Chem. 278 (50), 50355–50361. 10.1074/jbc.M305378200 12947118

[B64] Mohammadi K. Kometiani P. Xie Z. Askari A. (2001). Role of protein kinase C in the signal pathways that link Na +/K+-ATPase to ERK1/2. J. Biol. Chem. 276 (45), 42050–42056. 10.1074/jbc.M107892200 11562372

[B65] Mørk A. Geisler A. Mork A. (1993). Effects of minocycline on accumulation of cyclic AMP in cerebral cortex of rat. A comparison with lithium. Neuropharmacology 32 (8), 793–798. 10.1016/0028-3908(93)90188-9 8413842

[B66] Myles M. E. Fain J. N. (1994). Carbachol, but not norepinephrine, NMDA, ionomycin, ouabain, or phorbol myristate acetate, increases inositol 1, 3, 4, 5-tetrakisphosphate accumulation in rat brain cortical slices. J. Neurochem. 62 (6), 2333–2339. 10.1046/j.1471-4159.1994.62062333.x 8189237

[B67] Nguyen K. T. Buljan V. Else P. L. Pow D. V. Balcar V. J. (2010). Cardiac glycosides ouabain and digoxin interfere with the regulation of glutamate transporter GLAST in astrocytes cultured from neonatal rat brain. Neurochem. Res. 35 (12), 2062–2069. 10.1007/s11064-010-0274-4 20890657 PMC3002169

[B68] Nicotera P. Orrenius S. (1998). The role of calcium in apoptosis. Biometals. 11 (4), 375–382. 10.1023/A:1009226316146 10191500

[B69] O’brien W. J. Lingrel J. B. Wallick E. T. (1994). Ouabain binding kinetics of the rat alpha two and alpha three isoforms of the sodium-potassium adenosine triphosphate. Arch. Biochem. Biophys. 310 (1), 32–39. 10.1006/abbi.1994.1136 8161218

[B70] Okamoto Y. Kagaya A. Motohashi N. Yamawaki S. (1995). Inhibitory effects of lithium ion on intracellular Ca2+ mobilization in the rat hippocampal slices. Neurochem. Int. 26 (3), 233–238. 10.1016/0197-0186(94)00130-M 7787770

[B71] Olney J. W. (1986). Inciting excitotoxic cytocide among central neurons. Adv. Exp. Med. Biol. 203, 631–645. 10.1007/978-1-4684-7971-3_48 3024464

[B72] Orellana A. M. Leite J. A. Kinoshita P. F. Vasconcelos A. R. Andreotti D. Z. de Sa Lima L. (2018). Ouabain increases neuronal branching in hippocampus and improves spatial memory. Neuropharmacology 140, 260–274. 10.1016/j.neuropharm.2018.08.008 30099050

[B73] Ouabain (1932). Ouabain. Nat. Publ. Group 129, 88. 10.1038/129088e0

[B74] Panizza E. Zhang L. Fontana J. M. Hamada K. Svensson D. Akkuratov E. E. (2019). Ouabain-regulated phosphoproteome reveals molecular mechanisms for Na+, K+-ATPase control of cell adhesion, proliferation, and survival. FASEB J. 33 (9), 10193–10206. 10.1096/fj.201900445R 31199885 PMC6704450

[B75] Parreira G. M. Faria J. A. Marques S. M. S. Garcia I. J. P. Silva I. F. De Carvalho L. E. D. (2021). The γ-benzylidene digoxin derivative BD-15 increases the α3-Na, K-ATPase activity in rat Hippocampus and prefrontal cortex and no change on heart. J. Membr. Biol. 254 (2), 189–199. 10.1007/s00232-021-00173-2 33598793

[B76] Pierre S. v. Xie Z. (2006). The Na, K-ATPase receptor complex: Its organization and membership. Cell biochem. Biophys. 46 (3), 303–316. 10.1385/CBB:46:3:303 17272855

[B77] Pincus J. H. Lee S. (1973). Diphenylhydantoin and calcium. Relation to norepinephrine release from brain slices. Arch. Neurol. 29 (4), 239–244. 10.1001/archneur.1973.00490280051007 4728183

[B78] Prueitt R. L. Li W. Chang Y. C. Boffetta P. Goodman J. E. (2020). Systematic review of the potential respiratory carcinogenicity of metallic nickel in humans. Crit. Rev. Toxicol. 50 (7), 605–639. 10.1080/10408444.2020.1803792 33021439

[B79] Quintas L. E. M. Pierre S. v. Liu L. Bai Y. Liu X. Xie Z. J. (2010). Alterations of Na+/K+-ATPase function in caveolin-1 knockout cardiac fibroblasts. J. Mol. Cell. Cardiol. 49 (3), 525–531. 10.1016/j.yjmcc.2010.04.015 20451529 PMC5375041

[B80] Readhead C. Hood L. (1990). The dysmyelinating mouse mutations shiverer (shi) and myelin deficient (shimld). Behav. Genet. 20 (2), 213–234. 10.1007/BF01067791 1693848

[B81] Riganti C. Campia I. Kopecka J. Gazzano E. Doublier S. Aldieri E. (2011). Pleiotropic effects of cardioactive glycosides. Curr. Med. Chem. 18 (6), 872–885. 10.2174/092986711794927685 21182478

[B82] Sasaki T. Naka M. Nakamura F. Tanaka T. (1992). Ruthenium red inhibits the binding of calcium to calmodulin required for enzyme activation. J. Biol. Chem. 267 (30), 21518–21523. 10.1016/s0021-9258(19)36640-2 1383224

[B83] Satoh E. Nakazato Y. (1989). [3H]acetylcholine release and the change in cytosolic free calcium level induced by high K+ and ouabain in rat brain synaptosomes. Neurosci. Lett. 107 (1–3), 284–288. 10.1016/0304-3940(89)90832-X 2616040

[B84] Schatzmamn H. J. (1953). Cardiac glycosides as inhibitors of active potassium and sodium transport by erythrocyte membrane. Helv. Physiol. Pharmacol. Acta 11 (4), 346–354. 10.1038/cddis.2013.140 13142506

[B85] Schoner W. Scheiner-Bobis G. (2007). Endogenous and exogenous cardiac glycosides: Their roles in hypertension, salt metabolism, and cell growth. Am. J. Physiol. Cell Physiol. 293 (2), C509–C536. 10.1152/ajpcell.00098.2007 17494630

[B86] Shi M. Cao L. Cao X. Zhu M. Zhang X. Wu Z. (2019). DR-region of Na +/K + ATPase is a target to treat excitotoxicity and stroke. Cell Death Dis. 10 (1), 6. 10.1038/s41419-018-1230-5 PMC6315034 30584244

[B87] Somjen G. G. (2001). Mechanisms of spreading depression and hypoxic spreading depression-like depolarization. Physiol. Rev. 81 (3), 1065–1096. 10.1152/physrev.2001.81.3.1065 11427692

[B88] Stelmashook E. v. Weih M. Zorov D. Victorov I. Dirnagl U. Isaev N. (1999). Short-term block of Na+/K+-ATPase in neuro-glial cell cultures of cerebellum induces glutamate dependent damage of granule cells. FEBS Lett. 456 (1), 41–44. 10.1016/S0014-5793(99)00922-9 10452526

[B89] Sui L. Song X. J. Ren J. Ju L. H. Wang Y. (11992013). Intracerebroventricular administration of ouabain alters synaptic plasticity and dopamine release in rat medial prefrontal cortex. J. Neural Transm. 120 (8), 1191–1199. 10.1007/s00702-013-0973-5 23315013

[B90] Swanson P. D. Anderson L. Stahl W. L. (1974). Uptake of calcium ions by synaptosomes from rat brain. Biochim. Biophys. Acta 356 (2), 174–183. 10.1016/0005-2736(74)90281-8 4855335

[B91] Tata A. Velluto L. D’Angelo C. Reale M. (2014). Cholinergic system dysfunction and neurodegenerative diseases: Cause or effect? CNS Neurol. Disord. Drug Targets 13 (7), 1294–1303. 10.2174/1871527313666140917121132 25230223

[B92] Tian J. Cai T. Yuan Z. Wang H. Liu L. Haas M. (2006). Binding of Src to Na +/K + -ATPase forms a functional signaling complex. Mol. Biol. Cell 17 (1), 317–326. 10.1091/mbc.e05-08-0735 16267270 PMC1345669

[B93] v Blasi J. M. Cefia V. Gonzfilez-Garcia C. Marsal J. Solsona C. (1988). Ouabain induces acetylcholine release from pure cholinergic synaptosomes independently of extracellular calcium concentration. Neurochem. Res. 13 (11), 1035–1041. 10.1007/BF00973147 3237303

[B94] Valadares J. M. de M. Bajaj S. O. Li H. Wang H. L. Silva S. C. Garcia I. J. P. (2021). Cytotoxic effect of carbohydrate derivatives of digitoxigenin involves modulation of plasma membrane Ca2+-ATPase. J. Cell. Biochem. 122 (12), 1903–1914. 10.1002/jcb.30150 34553411 PMC8671332

[B95] Veldhuis W. B. van der Stelt M. Delmas F. Gillet B. Veldink G. A. Vliegenthart J. F. (2003). *In vivo* excitotoxicity induced by ouabain, a Na+/K+-ATPase inhibitor. J. Cereb. Blood Flow. Metab. 23 (1), 62–74. 10.1097/01.WCB.0000039287.37737.50 12500092

[B96] Volterra A. Trotti D. Tromba C. Floridi S. Racagni G. (1994). Glutamate uptake inhibition by oxygen free radicals in rat cortical astrocytes. J. Neurosci. 14 (1), 2924–2932. 10.1523/JNEUROSCI.14-05-02924.1994 7910203 PMC6577465

[B97] Whayne T. F. (0120). “Clinical use of digitalis: A state of the art review,” in American journal of cardiovascular drugs (Springer International Publishing), 18, 427–440. 10.1007/s40256-018-0292-1 30066080

[B98] Wiecien D. K. J. M. O’connor, O’connor L. T. Goetz B. D. Delaney K. H. Duncan I. D. (1998). Morphological and morphometric studies of the dysmyelinating mutant, the Long Evans shaker rat. J. Neurocytol. 27 (8), 581–591. 10.1023/a:1006922227791 10405025

[B99] Xiao A. Y. Wei L. Xia S. Rothman S. Yu S. P. (2002). Ionic mechanism of ouabain-induced concurrent apoptosis and necrosis in individual cultured cortical neurons. J. Neurosci. 22 (4), 1350–1362. 10.1523/jneurosci.22-04-01350.2002 11850462 PMC6757565

[B100] Xie Z. Askari A. (2002). Na+/K+-ATPase as a signal transducer. Eur. J. Biochem. 269 (10), 2434–2439. 10.1046/j.1432-1033.2002.02910.x 12027880

[B101] Yamazaki T. Akiyama T. Kitagawa H. KomakiF., Mori H. Kawada T. (2007). Characterization of ouabain-induced noradrenaline and acetylcholine release from *in situ* cardiac autonomic nerve endings. Acta Physiol. 191 (4), 275–284. 10.1111/j.1748-1716.2007.01749.x 17995575

[B102] Yuan Z. Cai T. Tian J. v Ivanov A. Giovannucci D. R. Xie Z. (2005). Na/K-ATPase tethers phospholipase C and IP3 receptor into a calcium-regulatory complex. Mol. Biol. Cell 16, 4034–4045. 10.1091/mbc.e05-04-0295 15975899 PMC1196317

[B103] Zhang S. Malmersjö S. Li J. Ando H. Aizman O. Uhlén P. (2006). Distinct role of the N-terminal tail of the Na, K-ATPase catalytic subunit as a signal transducer. J. Biol. Chem. 281 (31), 21954–21962. 10.1074/jbc.M601578200 16723354

[B104] Zhu Z. Tepel M. Neusser M. Zidek W. (1996). Low concentrations of ouabain increase cytosolic free calcium concentration in rat vascular smooth muscle cells. Clin. Sci. 90 (1), 9–12. 10.1042/cs0900009 8697711

[B105] Ziff O. J. Kotecha D. (2016). Digoxin: The good and the bad. Trends cardiovasc. Med. 26 (7), 585–595. 10.1016/j.tcm.2016.03.011 27156593

