# Supplementary material for: Effect of ouabain on calcium signaling in rodent brain: A systematic review of in vitro studies
Source: Front Pharmacol. 2022 Aug 29;13:916312. doi: 10.3389/fphar.2022.916312 (PMC9465813; doi:10.3389/fphar.2022.916312)
Supplement: Supplementary file 1 [file DataSheet1.docx]

Supplementary Material

**Supplementary table 1.** Eligibility criteria.

| P | Population | Studies in murine cells, tissues or model systems. |
| --- | --- | --- |
| E | Exposure | Exposure to cardiotonic steroids based on administered dose or concentration. |
| C | Comparasion | Compared with a non-exposed control group or base line parameter. |
| O | Outcome | Impact of different CTS on calcium signaling in neural substrates of murine models, including calcium transient and level, calcium binding proteins, calcium channel and pump activity and other related parameters. |

**Supplementary table 2.** Experimental conditions.

| **ARTICLE** | **EXPERIMENTAL CONDITIONS** |
| --- | --- |
| **SYNAPTOSOMES** | |
| Satoh and Nakazato, 1989 | Synaptosomes were prepared, according to the method of Hajós (1975). Change in cytosolic free calcium level [Ca2+] induced by ouabain was measured with spectrofluorometer using fluorescent indicator for Ca^2+^ (Fura-2 ). After 10 min of preincubation with Fura-2 at 37°C, ouabain was injected in the cuvette in the absence of extracellular Ca ^2+^.and recording for 6mim, approximately. |
| Adam-vizi and Ligeti, 1986 | Synaptosomes were prepared, according to the method of Hajós (1975). The pellet was suspended in 0-32 M-sucrose to give a final protein concentration of 20-25 mg/ml. After pre-incubation at 37 °C for 5 min, Ca^2+^ and ^45^Ca^2+^ together with depolarizing substances in 0-5 ml normal medium were added to provide a final concentration of 2 mM-Ca^2+^. To terminate ^45^Ca^2+^ uptake, the samples were incubated for 10 min at 37^0^C and radioactivity was determined in a liquid scintillation spectrometer. |
| Goddard and Robinson, 1976 | Synaptosomes were prepared by a modification of the method of White and Keen using discontinuous Ficoll gradient centrifugation. To calcium uptake experiments synaptosomes were equilibrated in the standard medium for 15 min at 37 °C, and then tracer quantities of ^45^Ca were added together with the OUA. The incubation was for 1 min. To time course of ^45^Ca retention by synaptosomes, the effiux of radioactivity into the perfusing medium was measured by liquid scintillation counting. |
| Swanson et al, 1974 | Synaptosomes were prepared by differential and density gradient centrifugation by the Bradford (1969) adaptation of the technique of Gray and Whittaker (1962). Uptake of calcium was carried out at 30 °C in oxygenated media. These studies were done in the presence of glucose as a primary substrate. Uptake of calcium was determined at l0 min after addition of 1.2 mM ^45^Ca, in the presence of OUA. |
| Blaustein et al, 1970 | Synaptosomes were prepared by differential and density gradient centrifugation by technique of Gray and Whittaker (1962). The synaptosomes were not centrifuged after preincubation, 2.4 mM ^45^Ca solution was added directly to the preincubation suspension (1.0 ml) to start the incubation that was for 2 min at 30^0^C. These studies were done in the presence of glucose. The ^45^Ca activity was assayed in a liquid scintillation counter, in the presence of OUA. |
| **SLICES** | |
| Bai et al., 2017 | Slice cultures were grown for 2-3 weeks before being used in experiments. Ouabain was added directly to the perfusate and the temperature was kept constant at 34^o^C. Ca^2+^ fluorescence imaging was measured using 50 μM Oregon Green BAPTA 1 in hybrid optical-magnetic resonance system. |
| Dietz et al., 2008 | The slices recover at 35°C for 60 min after cutting and were held at room temperature before being transferred to a recording chamber. Ca^2+^ measurements were made from individual CA1 pyramidal neurons. Neurons were impaled with sharp glass microelectrodes containing fluorescent indicator for Ca^2+^ (Fura-2 or Fura-6F) and cytosolic Ca^2+^ levels were monitored using a monochromator-based imaging system. Slices were maintained at 35°C during the experiment. |
| Basarsky et al., 1998 | For Ca^2+^ imaging experiments, slices were placed on solution of Fura-2 AM (10µM), supplemented with an additional 10 mM glucose for 2.5 h at a temperature of 25-30°C, transferred to room temperature (20-23°C) ACSF until used, and then were maintained at 33–34°C during the experiment. The fluorescent indicator for Ca^2+^, calcium orange, were used in astrocytes experiments. |
| Okamoto et al., 1994 | Hippocampal slices were incubated in gassed ACSF for 60 min, then in Fura-2/AM (10 µM) for 60 min (25^o^C). Fura-2 loaded slices were incubated in warmed ACSF for another 30 min and slices were maintained at 32°C during the experiment. [Ca^2+^]j was measured at 2 min intervals and the changes in [Ca^2+^]j were observed for 30 min. |
| Pincus et al., 1973 | Brain slices were weighed, sectioned and tissue weighing 90 to 100 mg was used. After 10 min in oxygenated Krebs-Ringer solution at 4^o^C, samples were exposed to ouabain and the tissue was transferred to vials. The medium consisted of 5 ml of oxygenated Krebs-Ringer solution and ^45^Ca^2+^. After washing, the tissue was homogenized in 1 ml of normal Krebs-Ringer solution. A 0.1 ml aliquot of homogenate was counted in a liquid scintillation spectrometer. |
| **CELL** | |
| Basseti et al, 2020 | The OPCs were pre-incubated for 24 h with ouabain. Cultures were incubated in artificial cerebrospinal fluid (ACSF) with 10 µM cell-permeable Ca^2+^ probe Oregon Green BAPTA 1 (OGB1) or Fura-2-AM, at 36^◦^C for 20 min. After staining, cells were washed twice with ACSF and transferred into a recording chamber for imaging experiments (as described in Fries et al, 2016). |
| Friess et al, 2016 | The OPCs were pre-incubated for 24 h with ouabain. Cultures were incubated in ACSF with 10 µM cell-permeable Ca^2+^ probe OGB1 or Fura-2-AM, at 36◦C for 20 min . After staining, cells were washed twice with ACSF and transferred into a recording chamber for imaging experiments. |
| Lomeu et al. 2003 | The [Ca^2+^]i in SN56 cells was measured for 35 min in the presence and absence of CaCl_2_, in a medium containing ouabain, using confocal microscopy and Ca^2+^ indicator Fluo-3. |
| Xiao et al, 2002 | Ratiometric fluorescence imaging were performed using Fura-2 AM (5µM), incubated for 60 min at 37◦C.The [Ca^2+^]i in neuronal cell bodies was measured for 90 min in the presence of ouabain. |
| Stelmashook et al, 1999 | The [Ca^2+^]i changes were monitored for 30 min after ouabain exposure of cell cultures Fluorescence data were obtained by monitoring Fluo-3. |
| Mark et al, 1995 | The [Ca^2+^]i was determined by ratiometric imaging with the Ca^2+^ indicator dye Fura 2- AM, immediately prior to, and 30 min following, exposure to ouabain. |
